# Application of Animal Models in Interpreting Dry Eye Disease

**DOI:** 10.3389/fmed.2022.830592

**Published:** 2022-02-01

**Authors:** Jun Zhu, Takenori Inomata, Kendrick Co Shih, Yuichi Okumura, Kenta Fujio, Tianxiang Huang, Ken Nagino, Yasutsugu Akasaki, Keiichi Fujimoto, Ai Yanagawa, Maria Miura, Akie Midorikawa-Inomata, Kunihiko Hirosawa, Mizu Kuwahara, Hurramhon Shokirova, Atsuko Eguchi, Yuki Morooka, Fang Chen, Akira Murakami

**Affiliations:** ^1^Department of Ophthalmology, Juntendo University Graduate School of Medicine, Tokyo, Japan; ^2^Department of Ophthalmology, Northern Jiangsu People's Hospital, Yangzhou, China; ^3^Department of Digital Medicine, Juntendo University Graduate School of Medicine, Tokyo, Japan; ^4^Department of Hospital Administration, Juntendo University Graduate School of Medicine, Tokyo, Japan; ^5^Department of Ophthalmology, Li Ka Shing Faculty of Medicine, The University of Hong Kong, Pokfulam, Hong Kong SAR, China

**Keywords:** dry eye, animal model, lacrimal gland, tear deficiency, evaporative, environmental stress, translational research, DED

## Abstract

Different pathophysiologic mechanisms are involved in the initiation, development, and outcome of dry eye disease (DED). Animal models have proven valuable and efficient in establishing ocular surface microenvironments that mimic humans, thus enabling better understanding of the pathogenesis. Several dry eye animal models, including lacrimal secretion insufficiency, evaporation, neuronal dysfunction, and environmental stress models, are related to different etiological factors. Other models may be categorized as having a multifactorial DED. In addition, there are variations in the methodological classification, including surgical lacrimal gland removal, drug-induced models, irradiation impairment, autoimmune antibody-induced models, and transgenic animals. The aforementioned models may manifest varying degrees of severity or specific pathophysiological mechanisms that contribute to the complexity of DED. This review aimed to summarize various dry eye animal models and evaluate their respective characteristics to improve our understanding of the underlying mechanism and identify therapeutic prospects for clinical purposes.

## Introduction

Dry eye disease (DED) is one of the most common ocular surface disorders affecting millions of people worldwide (1–3). Its symptoms range from irritation and light sensitivity to blindness in severe cases (4–6). The tear film is the most important component of the ocular surface in maintaining microenvironment stability and providing lubrication to the cornea, thus maintaining its refractive function (7–9). Factors that affect the production or quality of tear film may lead to DED; these include but are not limited to lacrimal gland impairment, inflammation, infection, systemic autoimmune conditions, and environmental stress (10–14). The pathogenesis of DED is complicated, and much remains unknown. Animal models of DED are essential to better investigate the mechanisms underlying this multifactorial condition, explore potential therapeutic targets, and identify factors that can accurately predict prognosis. Conventionally, DED is classified into two categories, namely tear-deficient and evaporative (15). Recent evidence from a translational research using animal models demonstrated that tear film dysfunction involves multiple risk factors, with ocular surface inflammation an important component, where the resident immune cells initiate and propagate alterations in the ocular surface microenvironment (16, 17). Herein, we reviewed various dry eye animal models from published literature according to different modeling methods. We will discuss their therapeutic targets and relative advantages and disadvantages in this translational research.

## Aqueous Deficient Dry Eye Types

### Lacrimal Gland Excision/Radiation Models

The surgical removal of the lacrimal gland in mice and rats is the most commonly reported model in DED research. Generally, the main procedures for mice lacrimal gland excision are as follows: after anesthetic administration, an incision is made to expose the extraorbital and/or intraorbital lacrimal gland with the aid of stereoscopic microscopy, and glands are generally excised and removed using micro-forceps and Vannas scissors without injuring surrounding blood vessels and nerves. All excisions are made ipsilaterally, usually on the right side, and only a sham surgery with cutaneous incision is performed on the contralateral side simultaneously (18, 19).

Principally, the excision of the primary lacrimal gland immediately reduces aqueous tear secretion. Compromised tear production manifesting with a reduced Schirmer's test score is the major outcome (20). This model can also be applied to other animals, such as dogs, rabbits, cats, and monkeys (21–24). The aforementioned models have demonstrated reduced Schirmer's test values and decreased basal tear production. However, vast differences in ocular surface anatomy and physiology between these models lead to inconsistency in the extent of affecting tear production, the duration of ocular surface complications, and reversibility. This warrants the selection of an appropriate animal specific to the needs of each study. For example, according to studies on the rat and mouse dry eye model (19, 25), the excision of the extra-orbital lacrimal gland reduces the tear volume and results in significant corneal epitheliopathy. However, decreased tear production does not necessarily manifest signs of ocular surface disease (26). Contrarily, the severity of dry eye symptoms may vary between genders. Mecum et al. (18) reported on a gender predilection that female mice were more susceptible to lacrimal gland excision-induced corneal damage. Regarding conjunctival changes resulting from the lacrimal gland removal, the evidence seems to vary among different animal modes. Stevenson et al. (19) reported increased T helper 17-cell frequencies in the conjunctiva and draining lymph nodes after extraorbital lacrimal gland removal in female C57BL/6 mice over 14 days. In contrast, Maitchouk et al. (21) concluded that the main lacrimal gland removal is not related to keratoconjunctivitis sicca.

External beam radiation therapy is a risk factor for DED following the treatment of head and neck cancers (27–29). Thus, it can be used to induce DED in animals. Hakim et al. (30) reported on a rabbit dry eye model using 15 Gy external beam radiation, which resulted in the functional impairment of the lacrimal gland and reduced tear production. Rocha et al. (31) introduced serial radiation doses at 8 or 6 Gy, delivered over 5 consecutive days, and successfully induced dry eye syndrome in mice. They observed a considerably lower production of tear secretion in the radiation group as well as a reduction in the epithelial thickness of the cornea, the absence of basal epithelium, and the thickening of corneal stroma at 10 days. However, these additional differences were transient and disappeared at 56 days post-radiation. Thus, radiation-induced dry eye models are reproducible and involve reversible alterations that provide a platform for mechanistic research into the treatment and prognosis of DED (32). The nuclear factor of activated T cells 5 plays a potential role, whereas α-lipoic acid exerts a protective effect on this radiation-induced model (33). However, compared with the previous simple lacrimal gland removal method, this method requires special radioactive equipment and poses certain risks and collateral damage.

### Blockage of Lacrimal Gland-Associated Nerve Pathways

The lacrimal nerve displays extensive sympathetic innervation, thus influencing tear production and composition (34). Therefore, researchers can develop aqueous-deficient models by blocking the afferent or efferent nerves associated with the lacrimal gland. Parasympathetic blockers are the first and foremost drugs. As a competitive antagonist of muscarinic acetylcholine receptors, atropine blocks the action of acetylcholine, thereby suppressing the function of the parasympathetic nervous system. In an albino rabbit dry eye model (35), atropine eye drops considerably reduced tear production and Schirmer's test scores within 2 days of onset. Aicher et al. (36) subcutaneously injected male Sprague-Dawley rats with 0.1% methyl atropine (1 mg/kg) twice daily for 2 days. Methyl atropine considerably reduced basal tear production compared with pretreatment baselines. This dry eye model can be implemented rapidly and easily; however, it is relatively simple and does not represent complicated pathophysiological processes in human dry eye syndrome.

Another method involves the use of neurturin-deficient (NRTN^−/−^) transgenic mice. Neurturin is a neurotrophic factor that regulates neuronal survival and function (37). Neurturin-deficient mice develop ocular surface inflammation similar to that observed in human DED. This transgenic mouse model substantially decreased aqueous tear production, tear fluorescein clearance, and corneal sensation (38). Moreover, it provided researchers with a method for better understanding the association between lacrimal gland denervation and ocular surface inflammation in DED. Nonetheless, its disadvantages are that the use and verification of this model are time-consuming.

### Autoimmune Disease-Associated Dry Eye Models

A specific class of mice shares common characteristics with autoimmune DED, owing to the presence of a specific mutation or gene-editing technique. They release autoreactive lymphocytes to attack their lacrimal glands, consequently resulting in tear secretion deficiency. They can be categorized as experimental autoimmune disease-associated dry eye models (Table 1).

**Table 1:** Autoimmune disease-associated dry eye models.

**Models**	**Modeling method**	**Modeling genre**	**Sex preference**	**Pathogenesis**	**Detectable serum antibodies**	**Pathogenic effects**	**References**
NOD mouse	Spontaneously developed	SS-like	Male	CD4^+^ Th1 cell infiltration of the lacrimal gland	Anti-thyroid Ab Anti-ss-A/Ro Anti-b adrenergic R Ab Anti-a-fodrin Ab Anti-M3 muscarinic R Ab	Pancreas, submandibular, and thyroid gland	(39)
NOD.B10.H2b mice	NOD mutant with an altered MHC region	SS-like	Male	Did not develop autoimmune diabetes, but displays lacrimal T-cell infiltration	Similar with NOD mouse	Similar to NOD mouse	(40)
MRL-1 pr/1pr mouse	Mutated Fas antigen	2nd-SS.	Female	Serological manifestations characteristic of SS and exhibit lacrimal gland infiltration, predominantly by CD4– T cells	Anti-ds DNA Ab Anti-ss DNA Ab Anti-gp70 Ab Rheumatoid factor Anti-SA/Ro Anti-SS-B/La	Sialadenitis Dacryoadenitis Kidney Joints blood vessels	(41)
Id3-deficient mice	Gene knockout	pSS-like	ND	T-cell-dominant lymphocyte infiltration in both lacrimal and salivary glands	Anti-SSA/Ro Anti-SSB/La antibodies	Reduced abilities to secrete tears and saliva	(42)
NFS/sld mouse	Spontaneous autosomal recessive mutation	pSS-like	Female	Lymphocytic infiltrates in exocrine glands are dominated by CD4^+^ T cells, with fewer CD8^+^ T cells and B cells	Anti-a-fodrin Ab	Inflammatory changes in the submandibular, parotid, and lacrimal glands	(43)
3d-Tx NFS/sld mouse	Thymectomy of NFS/sld mice at 3 days of age	pSS-like	Female	Thymectomy impairs the expansion of regulatory T cells	ND	Severe than NFS/sld mouse	(44)
CD25KO mice	Interleukin 2 receptor alpha gene knockout	SS-like disease	ND	Worsening of corneal surface parameters and an increase of CD4+ T cell infiltrating the cornea	Anti-RBC antibody	Age-dependent SS-like autoimmune lacrimal-keratoconjunctivitis, dacryoadenitis, and corneal epithelial disease	(45, 46)
PD knock-in mouse	p65 S276D knock-in mice	KCS or SS-like	ND	Dependent on NF-κB; TNFR1-independent corneal inflammation	ND	Genetic and independent of decreased lacrimal function; Dacryoadenitis	(47)
IQI/Jic mouse	CR-derived inbred strain	2nd-SS disease	Female	Focal lymphocyte infiltration and tissue destruction in the salivary glands (SG) and LG	Antinuclear autoantibodies	Salivary and lacrimal glands, pancreas, and lungs dysfunction	(48, 49)
Aly/aly mouse	Spontaneous autosomal recessive mutation	SS-like disease	No predilection	Chronic inflammatory cell (CD4+ T cell) infiltration in multiple organs	No detectable autoantibodies against nuclear components or salivary gland proteins	Cell infiltration in multiple organs, including the salivary and lacrimal glands, pancreas, skin, bones and lungs	(50)
TGF-b1 Knock out mouse	Gene knockout	SS-like disease	ND	Mainly CD4+ T cells infiltration	ND	Heart, lung, pancreas, lacrimal, salivary, and submandibular gland	(51, 52)

*KCS, keratoconjunctivitis sicca; ND, not determined; pSS, primary Sjögren's syndrome; and 2nd-SS, secondary Sjögren's syndrome*.

Sjögren's syndrome is a systemic autoimmune disease that causes secretory gland dysfunction (53). Several genetically modified mice have been used to mimic Sjögren's syndrome in dry eye studies. The non-obese diabetic (NOD) mouse model is mostly used for type 1 diabetes mellitus (54). NOD mice are susceptible to the spontaneous development of autoimmune insulin-dependent diabetes mellitus (55). Moreover, it facilitates investigating the influence of autoimmune processes on dry eye syndrome (56, 57). Lymphocytic infiltration leads to the degradation of extracellular matrix structures in the lacrimal gland of NOD mice (58). Ju et al. (39) reported that NOD mouse lacrimal glands displayed increased lymphocytic infiltration. Furthermore, they demonstrated substantially increased expression of major histocompatibility (MHC) II and interferon-γ in the lacrimal gland at 12 and 20 weeks. However, this model demonstrated a higher incidence of dacryoadenitis in male NOD mice than in females (59), thus suggesting the spontaneous autoimmune response may be modulated by sex steroids, particularly testosterone. Autoimmune lesions in this model involve autoreactive Th1 cell secretions, including interleukin (IL)-10 and IL-12. Sjögren's syndrome in the NOD mouse model is an interleukin-4, time-dependent, antibody isotype-specific autoimmune disease (60). Recently, Robinson et al. reported on a NOD-derived murine model (40), where NOD.B10.H2b mice, comprising MHC congenic to NOD, exhibited exocrine gland lymphocytic infiltration typical of Sjögren's syndrome-like disease and dysfunction observed in NOD mice, but without diabetes. Thus, the NOD.B10.H2b mouse model is considered interesting for studying primary Sjögren's syndrome.

The MRL*/1pr* mouse is a model of autoimmune arteritis, antiphospholipid syndrome, and systemic lupus erythematosus-like autoimmune syndromes (61–63). The *lpr* gene is a mutated *Fas* antigen that leads to lymphoproliferative disease (64). This model demonstrates anti-Ro/Sjögren's-syndrome type A and anti-La/Sjögren's syndrome type B autoantibody production, a characteristic manifestation of Sjögren's syndrome, besides exhibiting lacrimal gland infiltration, predominantly by CD4^+^ T cells (41). Unlike the NOD mouse model, the extent of inflammation is considerably greater in the lacrimal glands of female MRL/*lpr* mice than that in males (65). Furthermore, MRL/*lpr* mice develop glomerulonephritis, which is classic in systemic lupus erythematosus but rare in Sjögren's syndrome (66). Therefore, the MRL */1pr* mouse model is usually considered for secondary Sjögren's syndrome.

The inhibitor of DNA binding 3 (Id3) is an immediate early response gene involved in growth regulation and T-cell receptor-mediated T cell selection during its development (42). Id3-deficient mice develop lymphocyte infiltration in the lacrimal and salivary glands, reducing tear and saliva secretion and detectable anti-Ro and anti-La antibodies in the mouse serum. Similar to the clinical manifestations of primary Sjogren's syndrome, Id3-deficient mice serve as useful dry eye models for primary Sjogren's syndrome.

Conjunctival changes were also observed in autoimmune disease-related dry eye models. Using an autoimmune disease model mouse, BXSB/MpJ-Yaa, Hiraishi et al. (67) observed that goblet cell density in the conjunctiva epithelium decreased at 20 and 28 weeks compared to at 8 weeks. Wang et al. (68) reported spontaneous Sjögren-Like lacrimal keratoconjunctivitis in germ-free C57BL/6 mice, with significant goblet cell loss compared to conventional mice.

Researchers have reported other models, including the New Zealand Black and New Zealand White mouse (30, 35), the NFS/sld mouse (44), the IQI/Jic mouse (48, 69), the CD25 knockout mouse (45, 46), and the transforming growth factor β1 knockout mouse (51, 52). These autoimmune models affect the function of the lacrimal gland, eventually resulting in inadequate tear production. Depending on the characteristics of the specific model, they can be applied to different scenarios. Of all autoimmune dry eye models, the NOD mouse (39, 40) and the MRL-1 *pr/1pr* mouse model (41) are most commonly reported in scientific literature.

## Evaporative Dry Eye Models

### Environmental Stress-Induced Dry Eye Models

In addition to decreased tear production, DED can occur despite normal tear secretion in the context of significant environmental stressors. Numerous models utilize changes in the external environment, ventilation, humidity, or the forced exposure of the ocular surface to simulate evaporative DED.

Dursun et al. (70) introduced a desiccating environment by placing mice in a hood with a continuous airflow blower, with a flow rate of 300 ft/min at 7 s for 1 h, thrice per day for 4 days. Mice placed in the blower hood manifested the most severe ocular surface disease. Simsek et al. (71) used a model in which BALB/c male mice were exposed to an air fan inside a small compartment for 5 h per day for 3 days. The external environment considerably decreased the tear volume and increased corneal fluorescein and lissamine green staining scores. Moreover, the corneal subbasal nerve density was substantially damaged following exposure. Contrarily, several studies have introduced dry eye mouse models induced by air pollution particulate matter (PM), such as PM_10_ and urban particulate matter (UPM). PM_10_ impairs tear film function and destructs the structural organization of the ocular surface in mice. The topical administration of PM_10_ in mice induces ocular surface changes, similar to those induced by DED in humans (72). UPM exposure induces apoptosis in the corneal epithelium and decreases the number of goblet cells in the conjunctiva. Moreover, it affects the stability of the tear film by disrupting its mucin-4 layer (73). The advantage of these models, which simulate the real environment, is highly relevant to the development of environmentally-induced ocular surface diseases, including DED.

In another model, researchers introduced a lid retractor to prevent blinking. This model can be used in a short time and is easy to implement. It enables testing preventive and therapeutic strategies for DED (74). However, minimal changes in the ocular surface during a short preparation time limit its application in studying dry eye syndrome.

Other models exposed animals to low-humidity environments and continuous airflow. Chen et al. (75) established a murine model of DED using an intelligently controlled environmental system that maintained low humidity. Animals exposed to this environment exhibited decreased aqueous tear production, increased corneal fluorescein staining, and marked thinning and accelerated desquamation of the apical corneal epithelium compared with control eyes. The dry eye environment supposedly upregulated apoptosis on the ocular surface. Furthermore, biological and morphological changes in this model were similar to those in human DED. Barabino et al. (76) developed a controlled-environment chamber and confirmed that low humidity could substantially alter tear secretion, goblet cell density, and related ocular surface signs. Moreover, Nakamura et al. (77) combined a low-humidity environment, continuous airflow, and jogging board treatment, which mimicked both mental and physical stress, to induce abnormal tear dynamics and superficial punctate keratopathy, similar to that in humans.

### Meibomian Gland Dysfunction Models

Meibomian glands produce lipids, which are important components of the tear film (78). In physiological states, they prevent or lessen tear film evaporation, serve as the superficial protective layer, and stabilize the tear film by lowering surface tension. Lipid deficiency can lead to dryness of the ocular surface, damage to the conjunctiva and corneal epithelium, and an imbalance of the ocular surface microenvironment (79).

Jester et al. (80) introduced a meibomian gland dysfunction (MGD) model in 34 albino rabbits by topically applying 2% epinephrine twice daily, over 6 months to 1 year. Sixty-eight (56%) rabbits developed signs of MGD. The development and progression of MGD in rabbits appeared to correlate with increasing stratification and keratinization of the meibomian gland duct epithelium. Mishima et al. reported another rabbit MGD model by squeezing out meibomian gland contents and cauterizing the lid margin. Thus, a protective oily film layer could not form over the eyes of the treated animals, eventually leading to rapid tear evaporation. In another rabbit MGD model that used light cautery on meibomian gland orifices, researchers observed increased tear osmolarity in the presence of normal lacrimal gland function and ocular surface abnormalities, similar to that in keratoconjunctivitis sicca (81).

Other types of MGD models comprise transgenic mouse models, including X-linked anhidrotic-hypohidrotic ectodermal dysplasia (Tabby), apolipoprotein C1 transgenic mice, and ACAT-1-/- mice (82, 83). The meibomian glands were absent or abnormal in the aforementioned mice (Table 2). Tabby mice sequentially developed corneal epithelial defects, central corneal stromal edema, and corneal neovascularization 8–16 weeks following birth (91). Despite reduced tear film breakup time and tear evaporation times, tear secretion remained normal. This model is useful for identifying novel therapeutic agents for evaporative DED.

**Table 2:** Transgenic models of meibomian gland dysfunction.

**Models**	**Effect results**	**References**
ACAT-1–/–	Similar to dry eye syndrome in humans	(83)
TRAF6–/–	Defective development of epidermal appendixes	(84)
K14-Noggin	Replacement of meibomian glands in eyelids with hair	(85)
Smad4CKO	Hair follicles in place of the meibomian glands	(86)
Barx2–/–	Defective lacrimal gland morphogenesis, defects in meibomian gland	(87)
Klf5CN	Defective eyelids with malformed meibomian glands, the abnormal cornea loss of conjunctival goblet cells	(88)
Fatp4–/–	Abnormal development of both sebaceous glands and meibomian glands, specialized sebaceous glands of the eyelids	(89)
Fgfr2CKO	Significant meibomian gland acinar atrophy and clinical manifestations of MGD	(90)

*MGD, meibomian gland dysfunction*.

## Chemical-Induced Dry Eye Models

Researchers have used chemical substances, drugs, or biological agents to develop a class of lacrimal gland injury models. The scopolamine-induced dry eye model is most commonly used. Simsek et al. (92) assessed morphological changes in the corneal subbasal nerve plexus in wild-type mice following exposure to scopolamine. They observed decreased tear volume and shortened tear film breakup time (TFBUT). Confocal microscopy revealed substantially lower mean corneal subbasal nerve fiber density and reflectivity in the scopolamine-treated groups. Furthermore, the mean tortuosity and mean dendritic cell density were considerably higher in this model. Viau et al. (93) induced dry eye symptoms using scopolamine in 6-week-old female Lewis rats. Scopolamine was delivered via subcutaneously implanted osmotic pumps. TNF-α, IL-1β, and IL-6 mRNA levels increased with scopolamine treatment in both the conjunctiva and ex-orbital lacrimal glands. All animals exhibited unilateral or bilateral keratitis after 17 days. The scopolamine-induced dry eye model could serve as a stable model of moderate severity for dry eye studies.

Benzalkonium chloride (BAK) is the most frequently used preservative in eye drops. Researchers have consistently demonstrated its toxic effects on the ocular surface (94–96). It causes tear film instability, the loss of goblet cells, conjunctival squamous metaplasia and apoptosis, the disruption of the corneal epithelium barrier, and damage to deeper ocular tissues (97). In a rabbit BAK toxicity model, researchers demonstrated damage to the conjunctiva-associated lymphoid tissue via the topical application of BAK (98). In addition, Pauly et al. (99) developed a technical model that closely resembled the human ocular surface environment. They used fluorescence techniques conjugated with confocal microscopy on a 3-D reconstructed corneal epithelial model and observed an increase in apoptotic cells from the superficial to the deeper layers.

Botulinum toxin (BTX) is a potent toxin widely used in modern medicine (100) and has been used in various dry eye models. Park et al. (101) developed a mouse tear-deficient dry eye model without lacrimal gland inflammation by injecting BTX-B into the lacrimal gland. This model could effectively induce dry eye in mice 2 and 4 weeks following injection. The lacrimal structures were adequately maintained without significant T lymphocyte infiltration. Moreover, there are reports of a BTX-A-induced DED model in male C57BL/6 mice (102), which has also been demonstrated to be stable.

Researchers have also used other agents to establish different mouse, rat, or rabbit models, such as the injection of human recombinant interleukin-1 and concanavalin A into lacrimal glands and the oral administration of finasteride. Table 3 summarizes the chemical-, biological agent-, and drug-induced dry eye animal models.

**Table 3:** A summary of chemical-, biological agent-, and drug-induced dry eye animal models.

**Models**	**Modeling method**	**Demonstrated outcomes**	**References**
C57BL/6 female mice	Subcutaneous injection of scopolamine hydrobromide, an exposure to an air draft, and 30% ambient humidity	Tear-deficiency dry eye	(103)
Male Sprague-Dawley rats	Topically administered 10% N-acetylcysteine	Mucin-deficiency dry eye	(104)
Rabbit	Topically administered 0.1% benzalkonium chloride	Both aqueous tear and mucin deficiency	(105)
Rabbit	Burned with 50% trichloroacetic acid	Conjunctival goblet cells damage	(22)
Rabbit	Trichloroacetic acid-treated and/or the removal of nictitating membrane	Stable KCS model, similar to surgical LG removal	(106)
Rat	Subcutaneous implantation of scopolamine micro-osmotic pumps	Moderate dry eye	(93)
Wistar rats	Bilateral ovariectomy in female rats and oral finasteride; both male and female rats challenged	Tear deficiency Androgen deficiency dry eye	(107)
CBA/J mice	BTX-B injection into the LG	Mimic those in humans with non-SS, KCS	(108)
CBA/J mice	Transconjunctival injection of BTX-B into the lacrimal gland	Dry eye model without significant inflammatory cells infiltration	(109)
Female Wistar rats	Androgen deficiency dry eye model induced by finasteride	Androgen deficiency dry eye	(110)
Female BALB/c mice	A single injection of interleukin-1α into the lacrimal gland	A severe, but reversible inflammatory response in lacrimal gland acinar epithelial cells	(111, 112)

*BTX, botulinum toxin; KCS, keratoconjunctivitis sicca; LG, lacrimal gland; and SS, Sjögren's syndrome*.

## Discussion

**Figure 1:**
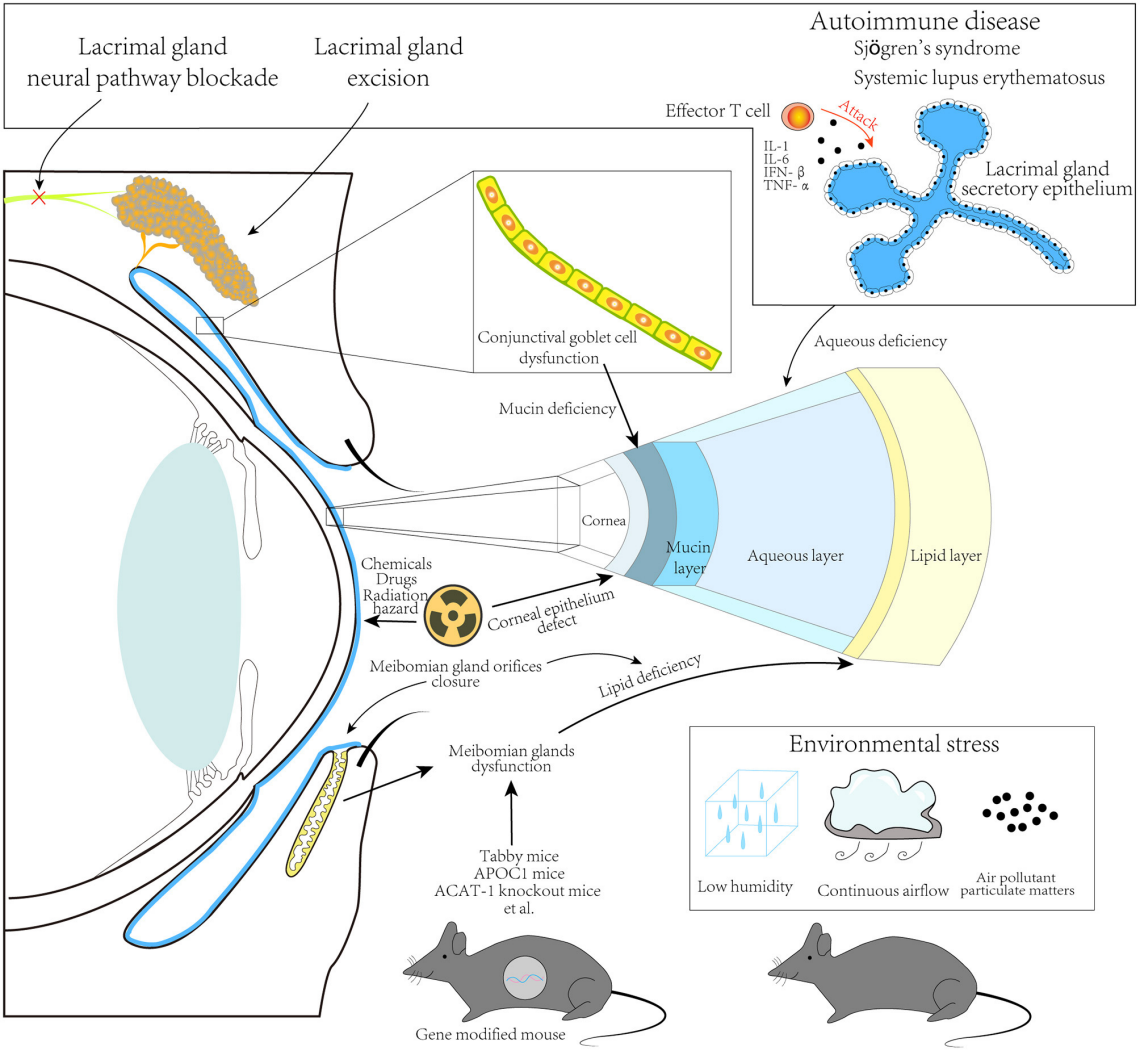
Principles for animal dry eye modeling. A schematic showing approaches to developing animal dry eye models, including major methods used in recently published studies, as described in this review. Lacrimal glands excision, neural pathway blocking, and autoimmune disease models, including Sjögren's syndrome and systemic lupus erythematosus, have been developed by targeting the lacrimal gland. These result in an aqueous deficiency in the tear film. Conjunctival goblet cell damage can result in mucin deficiency in the tear film. Further, chemicals, drugs, and radiation hazards mainly cause corneal epithelium damage. Some gene-modified mice can present with dysfunction or direct damage of the Meibomian glands, resulting in lipid deficiency in the tear film. Environmental stress due to changing humidity, controlling airflow, and/or introduction of air pollution particulate matter, could also be significant in animal dry eye modeling.

DED is a common ocular disorder that threatens the quality of life. Its symptoms vary from mild to severe, which are considered public health issues (113–115). The pathophysiologic mechanisms of DED comprise multiple factors that not only involve ocular surface inflammatory processes but are also related to systemic conditions (116, 117). Dry eye animal models for interpreting the underlying mechanisms are indispensable. We reviewed articles on such models, assessed their types, principles, and characters (Figure 1), and discussed their potential value for DED. A miscellaneous set of dry eye models has been created and provided to researchers. Their manifestations and severities vary according to the specific pathophysiological mechanism. Researchers should consider an appropriate model according to their objective.

The surgical removal of lacrimal glands, chemical- or drug-induced lacrimal damage, and challenging environments are convenient and effective methods for achieving aqueous deficiency and evaporative dry eye models. Generally, aqueous deficiency and evaporative models are the major classifications (15). Researchers have developed models based on these classifications according to different implementation methods (Table 4). They share common traits, including rapid symptom occurrence, short implementation time, and reproducibility in different animals. Moreover, they decrease tear secretion production, which is ideal for assessing the efficacy of different therapies. However, these models have different prerequisites. Specifically, a mouse model using lacrimal gland excision could induce severe aqueous deficiency; More dedicated surgical techniques are required. In addition, lacrimal gland removal may not fully reproduce the complete clinical dry eye phenotype and systemic diseases associated with DED, including Sjögren's syndrome, rheumatoid arthritis, and systemic lupus erythematosus because it is merely artificial. For chemical-induced models, the use of 1.0% atropine sulfate eye drops in albino rabbits rapidly provided the required symptoms on day 2 of treatment. In addition, Schirmer test scores, corneal fluorescein staining, and Ferning tests confirmed dry eye signs (35). However, the observations were conducted only until day 5, without additional data.

**Table 4:** Dry eye animal models.

**Animal models**	**Type**	**Species**	**Procedure**	**Experimental days**	**Effects to dry eye examinations**			**Pros**	**Cons**	**References**
					**Tear secretion volume**	**Tear film breakup time**	**CFS**			
Lacrimal gland excision	Aqueous deficiency	C57BL/6 WT mice	Exorbital and intraorbital lacrimal gland excision	14 days	↓16.6%	ND	↑*↑↑*	Inducing severe aqueous deficiency model	More surgically invasive	(118)
Radiation-induced model	Aqueous deficiency	female New Zealand rabbit	Radiation 15 Gy	3 days	↓	ND	ND	Objective surrogate parameters for radiogenic dysfunction	Requires special radioactive equipment	(30)
Drug-induced	Aqueous deficiency	Male New Zealand albino rabbits	1.0% atropine sulfate eye drop	2 days	↓↓	ND	↑	Producing the required symptoms rapidly	Only observed for 5 days, no longer time observation data	(35)
Drug-induced	Lacrimal Gland Denervation	Male Sprague-Dawley rats	192-IgG-saporin was microinjected into the lacrimal gland	3–4 weeks	No changes	ND	ND	Useful for exploring the mechanism underlying corneal hypoalgesia.	Microsurgery requirements; Normal basal tear production	(36)
Autoimmune model	Aqueous deficiency	NOD mice	Derived from the outbred Jcl:ICR line of mice	10–14 weeks	↓↓	61.43% 1~2 second	↑	Ideal model for autoimmune related DED	Discrepancies between preclinical studies and clinical outcomes	(54, 56, 119, 120)
Autoimmune model	Aqueous deficiency	MRL/lpr mice	Derived from the MRL/n mouse strain	16–18 weeks (female) 18–20 (male)	↓	ND	ND	A pivotal model for neurological SLE	Lack of data in the literature	(121, 122)
Autoimmune model	Aqueous deficiency	Id3-deficient mice	Gene knockout	8 weeks	↓	ND	ND	Ideal primary Sjogren's syndrome model	Technical challenges in gene knockout	(42)
Autoimmune model	Aqueous deficiency	IQI/Jic mice	Developed from outbred ICR mice	At least 9 months	ND	ND	ND	Model for secondary Sjogren's syndrome model	The age of onset limited its application	(48, 69)
Environmental stress	Evaporative dry eye model	Balb/c male mice	Exposed to an air fan 5 hours a day for 3 days	3 days	↓*↓↓*	↓*↓↓*	↑*↑↑*	Promising model to study the ocular surface and corneal nerve changes	Only male mice and acute alterations were assessed	(71)
Environmental stress	Evaporative dry eye mode	Rabbit	Eyes were held open with an eye specula	1–3 h	ND	ND	↑↑	Simply and short-term to implement	Not suitable for mechanism research	(74)
Meibomian gland dysfunction	Evaporative dry eye mode	New Zealand rabbit	Meibomian gland orifices were closed by electrical coagulation or light cautery	1–14 days	↓*↓↓* (Observed on day 1–3)	↓ (On day 3, 7, 14)	No difference	Suitable for MGD related dry eye research	Biochemistry and biophysics differences between rabbit and human meibum	(81, 123)

*CFS, corneal fluorescein staining; DED, dry eye disease; ICR, imprinting control region; MGD, meibomian gland dysfunction; ND, not determined; and SLE, systemic lupus erythematosus*.

*↑, increased; ↓, decreased*.

Evaporative DED models were developed by changing the feeding environment or imposing environmental stress on different animals. Animals were kept in a low-humidity environment or continuous airflow chambers for hours or days, which decreased tear film production, shortened the breakup time, and increased corneal fluorescence staining. The aforementioned environmental stress models are economical and easy to implement. Moreover, some studies focused on air pollution particulate matters, such as PM_2.5_, PM_10_ exposure, or simulating office working environments (72, 124, 125). These studies are valuable for investigating environmental factors of DED and the evaluation of related dry eye medicine therapies.

Evaporative dry eye models can be achieved by Meibomian gland dysfunction. One type involves the closure of the Meibomian gland orifices using electrical coagulation or light cautery. Lipid layer deficiency indirectly leads to aqueous layer evaporation. Usually, this type of model is combined with a low-humidity environment or rapid airflow to enhance evaporation. The abovementioned dysfunction of the Meibomian gland could provide tools for evaporative DED research; however, biochemical and biophysical differences between animal and human meibum limit its application. Other MGD models comprise transgenic models, such as ACAT-1, TRAF6, and Barx2 knockout mice, which exhibit the abnormal development of meibomian glands (83, 84, 87). These models are technically challenging, time-consuming, and expensive.

The short TFBUT-type dry eye model, with or without decreased tear secretion, highlights the importance of tear film stability in DED. Tear film stability is considered one of the important factors for understanding DED (126). Zhang Y et al. (127) developed a murine model based on graft-vs.-host disease (GVHD). Shimizu et al. (128) evaluated TFBUT in this GVHD-related model and observed significant differences in TFBUT, tear secretion, and corneal fluorescein scores between the syngeneic and GVHD groups from 9 to 12 weeks of age. Carpena-Torres et al. (129) reported on the topical instillation of 0.2% benzalkonium chloride for 5 consecutive days for establishing a dry eye model. The results demonstrated a significant difference in TFBUT before and after instillation; however, there was no difference in tear secretion. Different dry eye models are essential for understanding short TFBUT-type DED. Researchers have demonstrated a higher incidence of short TFBUT and concomitant keratoconjunctivitis in patients with thyroid eye disease (130, 131). Thus, these models facilitate understanding the etiology of short TBUT-type DED, particularly for patients clinically diagnosed with DED and normal tear secretion.

Dry eye models developed from autoimmune diseases provide insight into the immunopathogenic mechanisms of DED. NOD mice and MRL/*lpr* mice are typical systemic autoimmune disease models of Sjögren's syndrome, presenting multiple organ inflammatory lesions, including lacrimal gland damage that eventually results in aqueous deficiency. Both demonstrated significant lymphocytic infiltration of CD4^+^ T cells in the lacrimal gland. Dacryoadenitis revealed a higher incidence in male NOD mice than female mice. Compared with human Sjögren's syndrome, which is predominantly associated with female predilection, tear secretion was not profound in mouse models (57). MRL/*lpr* mice displayed more severe lacrimal gland inflammation in females than in males (65). Moreover, researchers observed a significant Th2 T cell response in the lacrimal gland of MRL/*lpr* mice, thus suggesting a mechanism different from the NOD mouse model (132). Nevertheless, these two models have been used for decades, with mature technology and sufficient research support, which are ideal dry eye models for DED.

This review summarized several major types of dry eye animal models and discussed their advantages and disadvantages in interpreting DED. This study had several limitations. It involved a limited number of animal species. The results of animal models differ considerably from human clinical manifestations. Moreover, they exhibit anatomical differences in the lacrimal gland system. Despite the variety of dry eye animal models, there are no widely homogeneous criteria for evaluating abnormalities of the ocular surface and the quality of tear film, thus making a model-wise comparison difficult.

## Conclusion

Researchers have used several dry eye animal models in translational research, each focusing on different pathophysiological mechanisms of DED. Of these models, lacrimal gland excision is the easiest and most practical method, and it is widely used in dry eye research. However, it is relatively simple as it only reflects the aqueous deficiency component of DED. Several dry eye models, such as NOD mice, MRL/*lpr* mice, and specific transgenic models, provide researchers with a better understanding of the underlying mechanisms of DED and ideas for the development of novel biological treatments. Models based on environmental stress and drug toxicity may be more feasible for studies on real-world risk factors for DED. However, DED in humans is usually multifactorial in nature and involves complicated pathophysiological and immune responses. Therefore, no single dry eye animal model can serve as the best tool for research.

## Author Contributions

TI: conceptualization and funding acquisition. TI and JZ: methodology, validation, and project administration. JZ: data curation and writing—original draft preparation. TI and KS: writing—review and editing. FC and AM: supervision. YO, KFujio, TH, KN, YA, KFujim, AY, MM, AM-I, KH, MK, HS, AE, and YM: review and suggestion. All authors have read and agreed to the publishing of the final version.

## Funding

This research was supported by JSPS KAKENHI Grant Numbers 20K09810 (TI), 20KK0207 (TI), 20K22985 (KFujim), 21K16884 (KFujim), and 21K20996 (HS). JZ was the recipient of a fellowship provided by the Japan-China Sasakawa Medical Fellowship program (Grant Number. 2018314).

## Conflict of Interest

The authors declare that the research was conducted in the absence of any commercial or financial relationships that could be construed as a potential conflict of interest.

## Publisher's Note

All claims expressed in this article are solely those of the authors and do not necessarily represent those of their affiliated organizations, or those of the publisher, the editors and the reviewers. Any product that may be evaluated in this article, or claim that may be made by its manufacturer, is not guaranteed or endorsed by the publisher.
